# An Identification Method for Rotor Direction Based on Charge Induction

**DOI:** 10.3390/s21041380

**Published:** 2021-02-16

**Authors:** Ronghui Chang, Limin Zhang, Jiaqun Lin, Feng Yan, Yong Chen

**Affiliations:** 1 School of Electronic Science and Engineering, Nanjing University, Nanjing 210023, China; mf1823002@smail.nju.edu.cn (R.C.); mf1923128@smail.nju.edu.cn (J.L.); fyan@nju.edu.cn (F.Y.); 2 Nanjing Zhongtuo Technology Co., Ltd., Nanjing 210000, China; yong.chen@njzhongtuo.com

**Keywords:** charge induction, rotor direction identification, high-input impedance sensor, correlation method

## Abstract

The detection of rotor motion is always key to ensure the normal operation of industrial sewing machines. This paper presents a novel method for rotor detection based on charge induction mechanism, which is suitable for industrial environments with high noise and electromagnetic radiation and is easy to install. Firstly, the principle of measuring rotor rotation based on charge induction is given. Then, the detection model of rotor direction identification based on two detection electrodes is established. Finally, details are given of the detection circuit design and the experiment that was carried out. The results show that the proposed method can effectively identify the noncontact rotor direction with and without occlusion, indicating that the method has excellent anti-interference capability. The accuracy of the method can be further improved by increasing the sampling rate and sampling points of the system.

## 1. Introduction

The monitoring and measurement of rotor movement is always essential in industry, aviation, precision machinery, and other fields. This includes industrial sewing machines that sew seatbelts, airbags, and technical textiles [1]. In order to ensure the working efficiency of industrial sewing machines, it is necessary to monitor the spinning state of the bobbin equipped with the bottom line during the sewing process. Usually, the bobbin steering affects the normal running of the bottom line. However, the space available to install a device to detect bobbin rotation is very small. Moreover, the sewing machine works in an industrial environment with high ambient noise and electromagnetic radiation.

The traditional mechanical tachometer, such as the centrifugal tachometer, can be used for preliminary detection of the rotor speed. In addition, a series of detection methods based on electricity, optics, and magnetism have been proposed [2,3,4,5]. Cheng Liu et al. proposed a rotational speed measuring system on the basis of a laser mouse sensor [6]. Didosyan et al. proposed a magneto-optical rotational velocity sensor based on an orthoferrite crystal [7], where magneto-optics provided the opportunity to combine the advantages of the optical methods with those of magnetic methods. Qian et al. put forward a noncontact method for the measurement of rotor speed and direction based on a quadrant photoelectric detector [8]. Fabian et al. developed a multiparameter, multisensor system for comprehensive electrical machine condition monitoring [9], where the fiber Bragg grating-based system can provide simultaneous monitoring of key parameters, including machine vibration, rotor speed, torque, spin direction, and temperature distribution, along the stator windings and on the rotor surface as well as the stator wave frequency. Iura et al. proposed an estimation method of rotational direction and speed according to the characteristic of an alternating current (AC) machine [10]. Digital image processing and vision system have also been used to detect rotor motion [11,12,13,14]. For example, a novel nonprojection fringe vision-based system simply composed of an artificial linearly varying-density fringe pattern as a sensor and a high-speed camera as a detector was proposed to realize the measurement of instantaneous rotational speed (IRS) in [15]. Guo et al. realized vision-based measurement for rotational speed by improving the Lucas–Kanade template tracking algorithm [16]. In recent years, acoustic methods for rotor research have also been presented [17]. Mata-Contreras et al. proposed a strategy to measure rotation speed and detect the motion direction in contactless angular velocity sensors operating at microwave frequencies [18].

However, there are some limitations in the detection methods proposed above. Optical methods, for example, are susceptible to background light and dust. The performance of methods based on the electromagnetic principle is easily affected by electromagnetic interference, so it is not suitable for industrial applications. Detection devices based on the image processing and vision system are complex in structure, require regular maintenance, and are high cost. Therefore, a rotation speed measurement method based on electrostatic sensor has been proposed [19,20]. In order to improve the performance of the electrostatic sensor in detecting rotation speed, Li et al. utilized a strip of predetermined material stuck on the rotational shaft that will accumulate charge to obtain a strong periodical signal [21]. The measurement method based on electrostatic sensor is not affected by dust deposition and electromagnetic interference, has a simple structure, and is low cost. However, the detection electrode in this method needs to be placed around the rotor and electrostatic shielding is added, which is difficult to install due to the lack of physical space in industrial sewing machines. Moreover, the rotor direction cannot be detected in this method.

A rotor with an uneven surface will generate a low-frequency electric field as it rotates. Our research group has been committed to carrying out application research in different directions based on the charge induction principle and has made good progress in hand motion direction recognition and intelligent light control in previous works [22,23]. Therefore, this paper proposes a rotor measurement method based on charge induction, which can realize rotor motion state monitoring and direction identification. Compared with the existing methods, the rotor detection method based on charge induction is characterized by easy installation and anti-influence of magnetic field, illumination, and dust. The rest of the paper is organized as follows. Firstly, the principle of measuring rotor rotation based on charge induction is given. Then, the detection model of rotor direction identification is established. Finally, the results of an experiment carried out to verify the effectiveness of the detection method with and without occlusion are discussed.

## 2. Detection Principle

### 2.1. Charge Detection Model for Rotor

When a rotor is in rotational motion, its surface becomes charged due to the friction with dust or particles in the surrounding environment. The charge on the rotor can create an electric field in space, which generates an induced charge on the conductor placed in the field. The relationship between the induced charge on the conductor surface and the electric field intensity can be expressed by [24]

(1) $$Q={∯}_{A}\varepsilon E•dA$$

where *Q* is the induced charge on the surface of the conductor, *E* is the electric field intensity, and *A* is the surface area of the conductor.

Assuming that the surface of the rotor is completely uniform, the distribution of the electric field generated by the rotating object will not change at any time, nor will the induced charge on the conductor surface. If a hole (or bump) is added to the edge of the rotor, the surface of the rotor will become uneven. As a rotor with a hole (or bump) rotates at a specific frequency, the induced charge on the electrode surface will periodically change. In order to simplify the detection model, the rotor rotation can be equivalent to the circular motion of the hole (or bump), and the hole (or bump) is further approximately considered as a rotating object with charge *Q*_0_.

**Figure 1:**
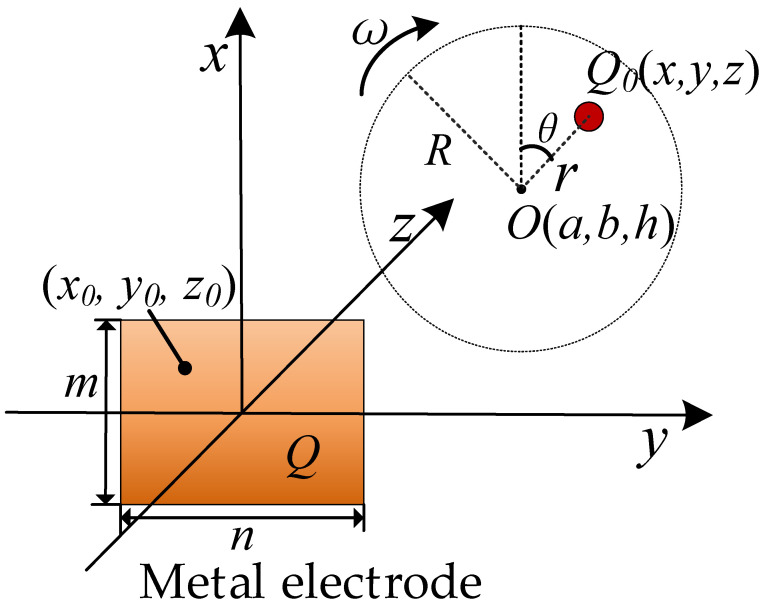
The principle of measuring rotor rotation based on charge induction.

The charge detection model for rotor is shown in Figure 1, where the Cartesian space coordinates is established. The charge *Q*_0_ rotates around the point *O* (*a*, *b*, *h*), and its trajectory is parallel to the *x–y* plane with the distance *h*. *Q* is the induced charge amount on the metal electrode produced by the charge *Q*_0_. The length and width of the metal electrode are *m* and *n*, respectively. The origin of the coordinate system is the center of the metal electrode, and the axes *x* and *y* are located on the plane of the metal electrode.

If the coordinate of charge *Q*_0_ is (*x*, *y*, *z*), the electric field intensity at the position (*x*_0_, *y*_0_, *z*_0_) on the metal electrode surface can be calculated as

(2) $$E_{\left(x_{0},y_{0},z_{0}\right)}=\frac{1}{4\pi \varepsilon }\frac{Q_{0}}{{\left(x-x_{0}\right)}^{2}+{\left(y-y_{0}\right)}^{2}+{\left(z-z_{0}\right)}^{2}}e_{r}$$

According to Equations (1) and (2), the induced charge *Q* on the metal electrode can be expressed as

(3) Q(Q0,x,y,z)=∯A−14πQ0z[(x−x0)2+(y−y0)2+(z−z0)2]32•dA=(-Q0/4π){arctan[(n/2−y)(m/2−x)/z(m/2−x)2+(n/2−y)2+z2]+arctan[(n/2+y)(m/2−x)/z(m/2−x)2+(n/2+y)2+z2]+arctan[(n/2−y)(m/2+x)/z(m/2+x)2+(n/2−y)2+z2]+arctan[(n/2+y)(m/2+x)/z(m/2+x)2+(n/2+y)2+z2]}

**Figure 2:**
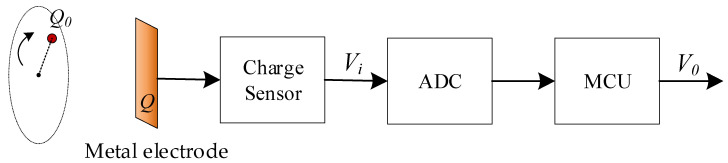
The process of rotor detection.

In order to intuitively observe the change process of the induced charge on the metal electrode, the detection system shown in Figure 2 is used for charge detection. The induced charge *Q* is first converted to voltage *V_i_* using a charge-to-voltage convertor with input capacitor *C_i_*. Then, the voltage *V_i_* is digitized by an analog-to-digital converter. Finally, a microprocessor is used to transmit the digital signal to the computer for further processing and analysis.

The output signal *V_o_* of the detection system is thus determined from

(4) $$V_{0}=\frac{Q(Q_{0},x,y,z)H}{C_{i}}$$

where *H* is the system gain. For the detection model described in Figure 1, it is assumed that the charge *Q*_0_ rotates around the point *O*(*a*, *b*, *h*) with angular velocity *ω*, initial phase *θ*, and radius *r*. Then, the coordinates of charge *Q*_0_ can be expressed as

(5) $$\left\{ \begin{array}{l} x=a+r\cos(\omega t+\theta )\\ y=b+r\sin(\omega t+\theta )\\ z=h \end{array}\right.$$

In Equation (5), if *a* = *b* = 0, that is, the center of the rotor is directly opposite the center of the metal electrode, then the induced charge *Q* obtained from Equation (3) will be a certain value, which can be expressed as

(6) $$Q=-\frac{Q_{0}}{4\pi }\frac{mn}{\sqrt{r^{2}+h^{2}}}$$

**Figure 3:**
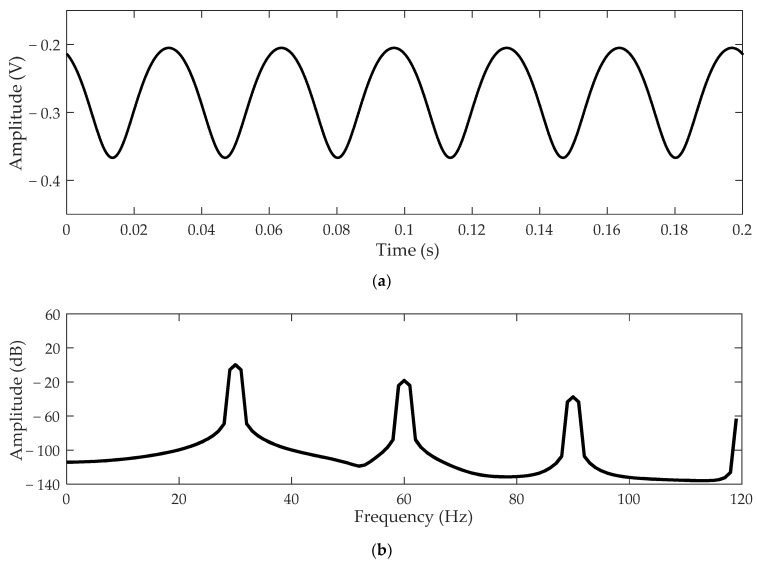
The simulation waveform of the output signal *V_o_*: (**a**) time-domain waveform; (**b**) frequency-domain waveform.

At this time, the charge detection system cannot reflect the periodic motion of the rotor, so it is necessary to ensure that *a* and *b* are not equal to zero at the same time. Supposing *Q*_0_ = 10^−10^ C, *C_i_* = 10 pF, *H* = 6, *a* = 2 cm, *b* = 3 cm, *h* = 4 cm, *m* = 1.5 cm, *n* = 1.5 cm, *r* = 0.8 cm, *ω* = 60π rad/s, and *θ* = π/2, the simulation waveform of voltage *V_o_* obtained through Equations (3)–(5) is shown in Figure 3. Figure 3a is the time-domain waveform of the output signal. It can be seen that the output signal *V_o_* is periodic due to the rotational motion of the rotor. Figure 3b shows the frequency-domain waveform of the output signal. There is an obvious signal of 30 Hz on the spectrum curve, which is exactly equal to the rotor speed. In addition, for other shapes of electrodes, such as circular electrodes, the calculation can be made by dividing them into smaller square electrodes. The amplitude of the output signal is mainly related to the electrode area rather than the shape. The larger the electrode area, the more the induced charge generated on the electrode.

**Figure 4:**
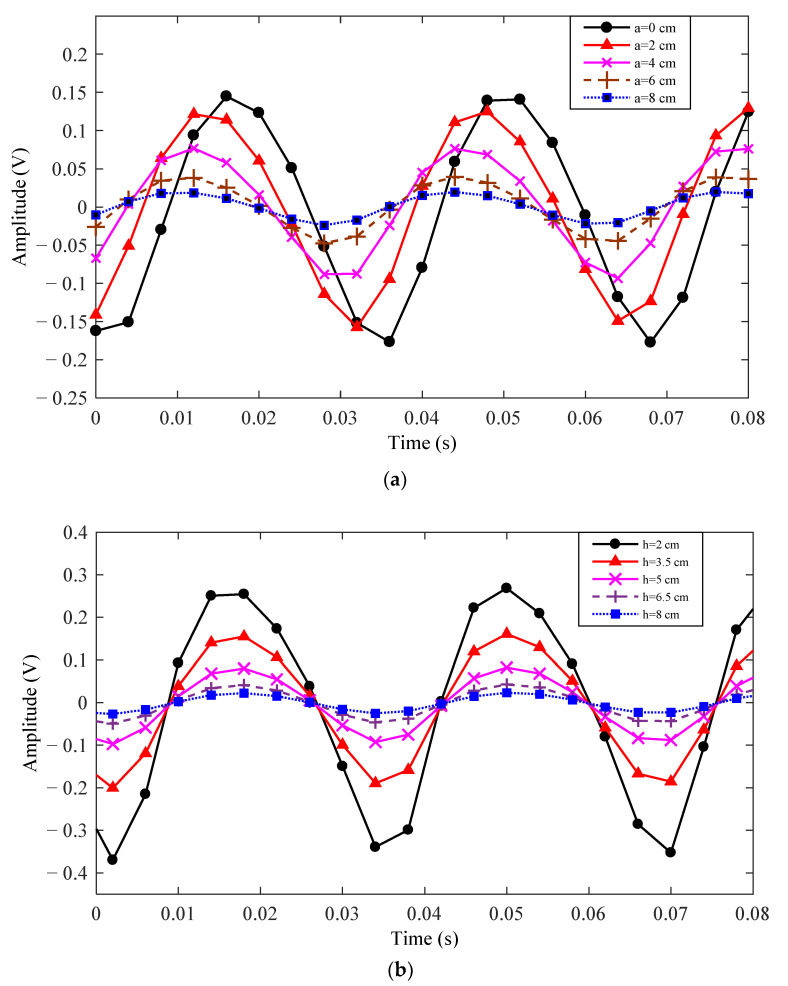
*V_o_* as functions of *a* and *h*: (**a**) *V_o_* as a function of *a* for *b* = 3 cm, *h* = 4 cm; (**b**) *V_o_* as function of *h* for *a* = 2 cm, *b* = 3 cm.

Figure 4 shows the relationship between the position of the rotor relative to the metal electrode and the AC component of the output signal *V_o_*. When *b* and *h* remain unchanged, the output signal *V_o_* as a function of the distance *a* of the rotor center relative to the metal electrode is shown in Figure 4a. When *a* and *b* remain unchanged, the output signals obtained with different vertical distances *h* are shown in Figure 4b. It can be seen that the increase of distance *a* and *h* can both lead to the amplitude decrease of *V_o_*. However, distance *a* will affect the zero crossing point of the output signal *V_o_*, while distance *h* will have no effect on it. It should be noted that the effect of parameter *b* on the output signal *V_o_* is similar to that of parameter *a*. 

### 2.2. Rotor Direction Identification Model

**Figure 5:**
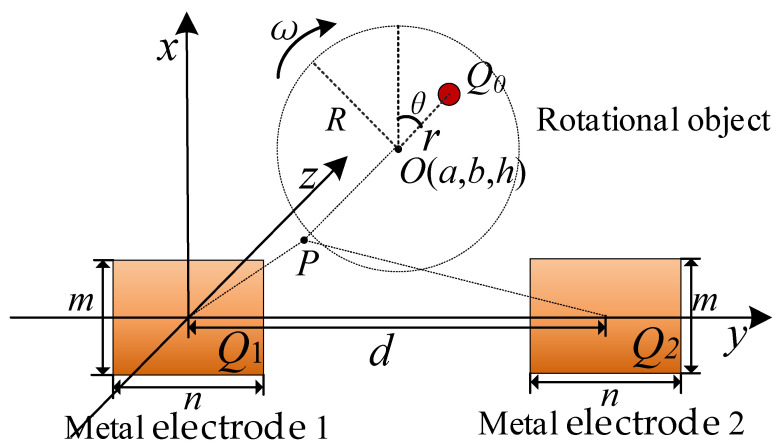
The detection model of rotor direction identification based on two detection electrodes.

In order to recognize the rotational direction of rotor, two metal electrodes of the same size are used to detect the induced charge, as shown in Figure 5. The plane of the two electrodes and the rotor surfaces are both placed parallel to the *x–y* plane, and *d* is the distance from the center of metal electrode 1 to the center of metal electrode 2. *Q*_1_ and *Q*_2_ are the induced charge amount on metal electrode 1 and metal electrode 2 produced by charge *Q*_0_. 

The coordinates of charge *Q*_0_ (*x*, *y*, *z*) relative to metal electrode 2 can be expressed as

(7) $$\left\{ \begin{array}{l} x=a+r\cos(\omega t+\theta )\\ y=b+r\sin(\omega t+\theta )-d\\ z=h \end{array}\right.$$

**Figure 6:**
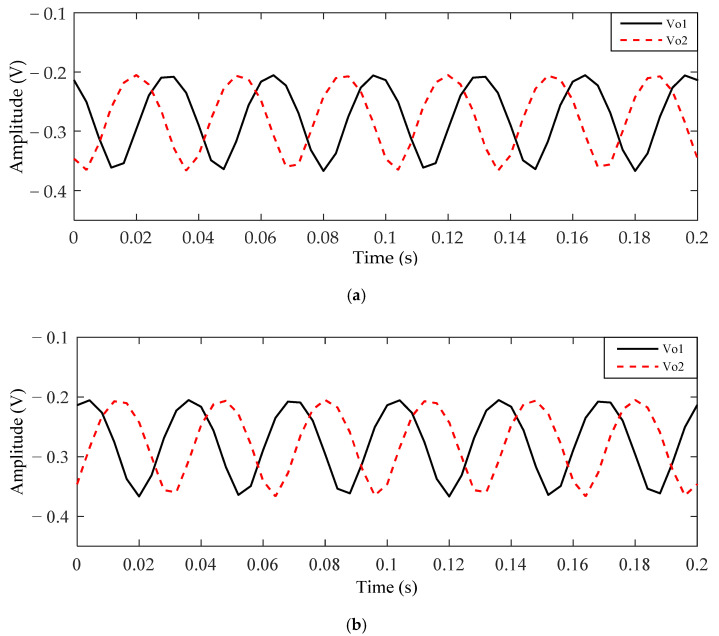
The ideal output signals of *V*_o1_ and *V*_o2_ for *d* = 6 cm: (**a**) signals *V*_o1_ and *V*_o2_ obtained when the rotor rotates clockwise; (**b**) signals *V*_o1_ and *V*_o2_ obtained when the rotor rotates anticlockwise.

Then, the output signal *V*_o1_ of metal electrode 1 can be obtained from Equations (3)–(5), and the output signal *V*_o2_ of metal electrode 2 can be obtained from Equations (3), (4) and (7). Supposing that the center of the two electrodes is symmetrical to the center of the rotor and the distance *d* between the centers of the two electrodes is equal to 6 cm, the output signals of the two metal electrodes can be obtained, as shown in Figure 6. If the rotor rotates clockwise, the ideal output waveform of *V*_o1_ and *V*_o2_ are given in Figure 6a. Similarly, Figure 6b shows the ideal output waveform of *V*_o1_ and *V*_o2_ obtained when the rotor rotates anticlockwise. During the rotation of the rotor, the hole (or bump) passes through the two metal electrodes, so the amplitude of the signals obtained from the two metal electrodes reaches the peak value successively. When the rotor rotates clockwise, the output signal of metal electrode 1 lags behind that of metal electrode 2. On the contrary, when the rotor rotates anticlockwise, the output signal of electrode 1 is ahead of that of electrode 2. Therefore, the rotation direction of the rotor can be identified by the phase relationship between the two output signals. It must be noted that the centers of the two metal electrodes are not in a straight line with point *P*, which is the projection point of the rotor center in the *x–y* plane; otherwise, the phase relationship between the two signals in the two rotation directions will be the same. The theoretical phase difference between the two output signals is equal to the angle between the centers of the two metal electrodes and point *P*. 

**Figure 7:**
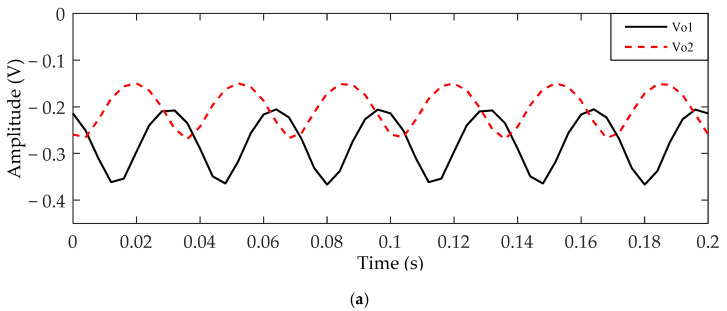

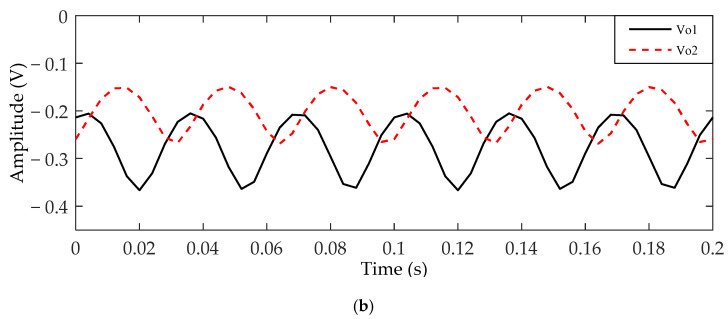
The ideal output signals of *V*_o1_ and *V*_o2_ for *d* = 7 cm: (**a**) signals *V*_o1_ and *V*_o2_ obtained when the rotor rotates clockwise; (**b**) signals *V*_o1_ and *V*_o2_ obtained when the rotor rotates anticlockwise.

When the distance *d* between the two electrodes is equal to 7 cm, the simulation output waveform of *V*_o1_ and *V*_o2_ are shown in Figure 7. It can be seen that although the signal amplitude and direct current (DC) bias of *V*_o1_ and *V*_o2_ are different, the phase relationships of *V*_o1_ and *V*_o2_ in the two rotation directions are still the same as that discussed above.

In order to analyze the phase relationship between the output signals obtained from the two electrodes, the phase difference can be calculated using the correlation method. The cross-correlation functions of two signals *A*(*t*) and *B*(*t*) in the time interval *T* can be given by

(8) $$R_{AB}(\tau )=\int_{0}^{T}A(t)B(t+\tau )dt$$

where the cross-correlation functions *R_AB_* reaches the maximum value for the same phase of two signals *A*(*t*) and *B*(*t*). Therefore, when *R_AB_* is the maximum, the time delay of *B*(*t*) with respect to *A*(*t*) is time *τ*. When the time delay *τ* is larger than 0, the phase of *B*(*t*) leads *A*(*t*). When the time delay *τ* is less than 0, the phase of *B*(*t*) lags *A*(*t*). When signals *A*(*t*) and *B*(*t*) are discrete signals, Equation (8) can be expressed as

(9) $$r_{AB}=\sum_{n=0}^{N}A(n)B(n+m),m=-N,-N+1,\ldots ,n=N-1,N$$

where *N* is the length of signals *A*(*n*) and *B*(*n*). Assume that the maximum value of *R_AB_* is obtained at the sampling point *M*. The time delay *τ* between *A*(*n*) and *B*(*n*) can be calculated by

(10) $$\tau =(M-N)/f_{s}$$

where *f_s_* is the sampling rate of signal *A*(*n*) and *B*(*n*). In order to further study the phase relationship between *A*(*n*) and *B*(*n*), the time delay is converted into phase difference:

(11) $$\Delta \theta =\frac{\tau }{f_{0}}\times 360^{\circ }$$

where *f*_0_ is the frequency of signal *A*(*t*) and *B*(*t*). To intuitively distinguish the phase relation between *V*_o1_ and *V*_o2_ shown in Figure 6, the value of phase difference Δ*θ* should be limited in the range from −180° to 180°. If the Δ*θ* is beyond the limit, the new phase difference Δ*θ’* can be calculated as follows:

(12) $$\Delta \theta ’=\left\{ \begin{array}{l} \Delta \theta -360^{\circ }\times ([\frac{\Delta \theta -180^{\circ }}{360^{\circ }}]+1),\Delta \theta >180^{\circ }\\ \Delta \theta \\ \Delta \theta +360^{\circ }\times ([\frac{\Delta \theta +180^{\circ }}{360^{\circ }}]+1),\Delta \theta <-180^{\circ } \end{array}\right.$$

where $\left[\frac{\Delta \theta \pm 180^{\circ }}{360^{\circ }}\right]$ is the largest integer less than $\frac{\Delta \theta \pm 180^{\circ }}{360^{\circ }}$. Thus, the phase difference between *V*_o1_ and *V*_o2_ shown in Figure 6 can be obtained. The phase difference in the clockwise direction is equal to 115.2°, and the phase difference in the anticlockwise direction is equal to −115.2°. Similarly, the phase difference in Figure 7 can be calculated. The phase difference is 129.6° in the clockwise direction and −129.6° in the counterclockwise direction. Therefore, the rotation direction of the rotor can be identified by the phase difference in comparison with 0.

## 3. Experimental Results and Discussion

**Figure 8:**
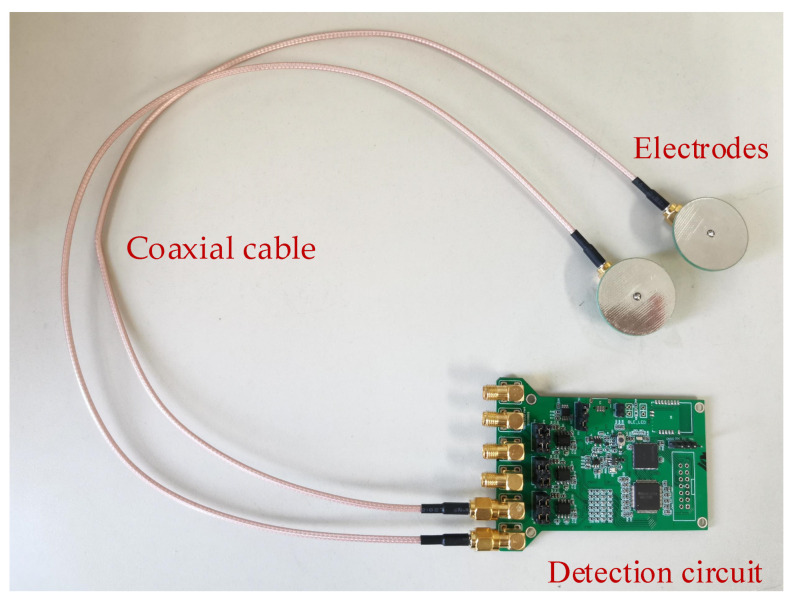
The detection circuit of the infrared system.

**Figure 9:**
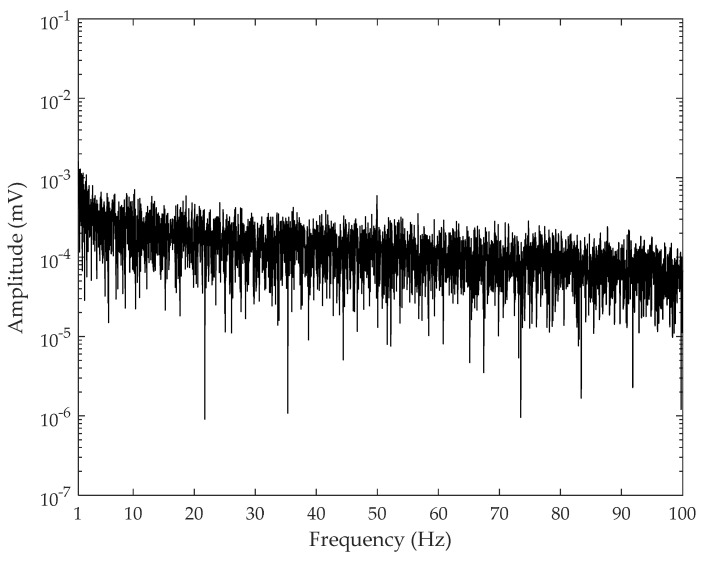
The input referred voltage noise in frequency domain.

**Figure 10:**
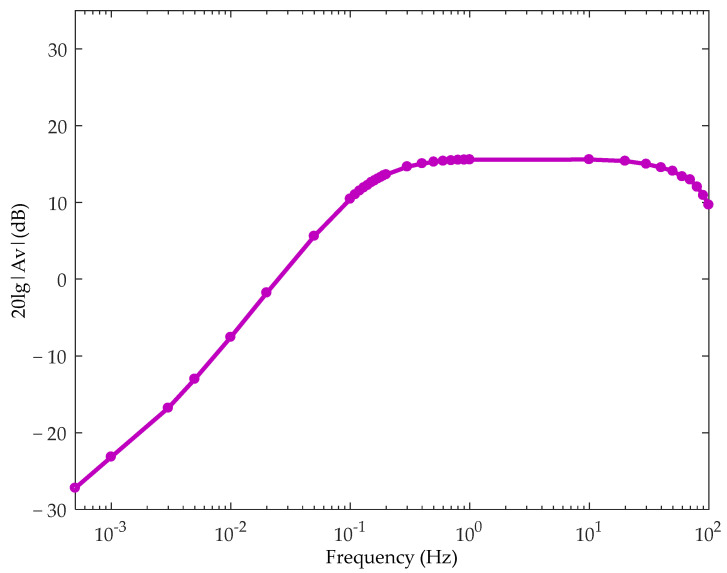
Amplification frequency response of the detection circuit.

The detection circuit used in the following experiment is shown in Figure 8, including the charge sensor, analog-to-digital converter (ADC) module, and microprocessor module, as mentioned in Figure 2. The charge sensor was the ultrahigh-input impedance circuit proposed by our group [25]. The ADC module was implemented with 24-bit ADS1298, and the microprocessor was MSP430F5528. The collected data was transmitted through serial port to the collection computer with a developed user interface program, and the input port of the whole module was connected to two detection electrodes through coaxial cable. The electrodes were circular metal plates with a diameter of 4 cm and tin plating on their surface. The input referenced short-circuit noise of the detection circuit is shown in Figure 9, where the noise for 1–100 Hz bandwidth was less than 1.5 μV. Figure 10 shows the frequency response diagram of the detection circuit. The system had a band-pass characteristic with a cut-off frequency of 0.15 and 75 Hz, and the gain at 30 Hz was about 15 dB.

The minimum and maximum charges that can be accurately detected by the proposed rotor direction identification system were determined by system performance parameters, such as system noise and the reference voltage of the ADC module. Firstly, the useful input signal must theoretically be larger than the input reference noise to ensure effective signal acquisition. However, in order to obtain a high signal-to-noise ratio, the minimum input voltage *V_i_* is generally required to be greater than five times the system noise. Secondly, considering that the saturation of the output signal will lead to identification failure, the maximum output voltage *V*_0_ should be guaranteed to be less than 90% of the reference voltage ±2.4 V of the ADC module with 15 dB gain. Therefore, the input voltage of the circuit designed in the experiment should be less than 385.7 mV and larger than 7.5 μV. Considering the system input capacitor *C_i_* 10 pF, the amount of induced charge on the metal electrode detected in the system ranged from 7.5 × 10^−17^ C to 3.86 × 10^−12^ C.

### 3.1. Experimental Condition

**Figure 11:**
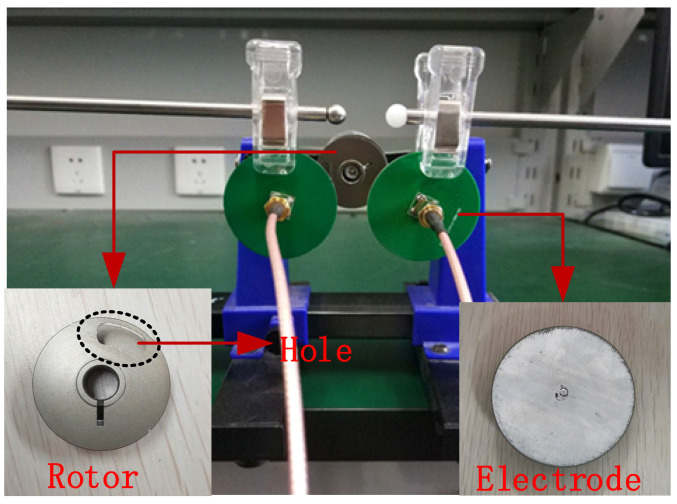
The experimental system for rotor motion.

**Figure 12:**
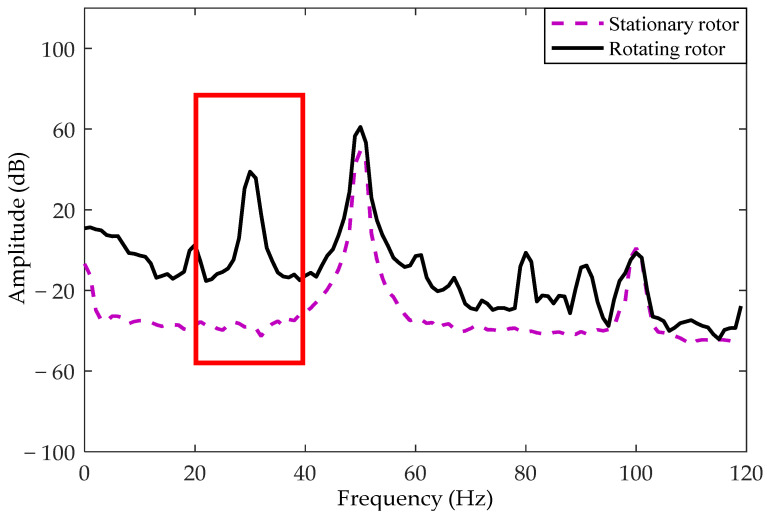
The output signal spectrum under both stationary and rotating conditions.

Figure 11 shows the experimental equipment for rotor detection. In the experiment, a hole was added at the edge of the rotor with a radius *R* of 1.5 cm, where the distance *r* between the hole center and the rotor center was set as 0.8 cm. The rotor was driven by a DC motor, and its rotation speed was adjusted by changing the amplitude of the DC voltage. The direction of the rotor was adjusted by changing the positive and negative poles of the power supply. Two electrodes were placed in the vicinity of rotating equipment being monitored as mentioned in Figure 5. The position of the rotor center was set as *a* = 2 cm, *b* = 3 cm, and *h* = 4 cm, and the center-to-center spacing between the electrodes was 6 cm. The experimental parameters were consistent with the simulation parameters except the charge value and initial phase. In addition, the area of the circular electrode with a diameter of 4 cm used in the experiment was slightly larger than that used in the simulation. The sampling rate of the system was selected to be 250 Hz. Finally, the output signal transmitted by the microprocessor was collected with the developed user interface program. The spectral curves of the output signal obtained by one of the electrodes are shown in Figure 12 under both stationary and rotating conditions of the rotor. Compared to the signal for rotor at rest, there was a significant 30 Hz signal for rotor rotation, which corresponded to the rotation frequency. 

### 3.2. Signal Processing and Analysis

**Figure 13:**
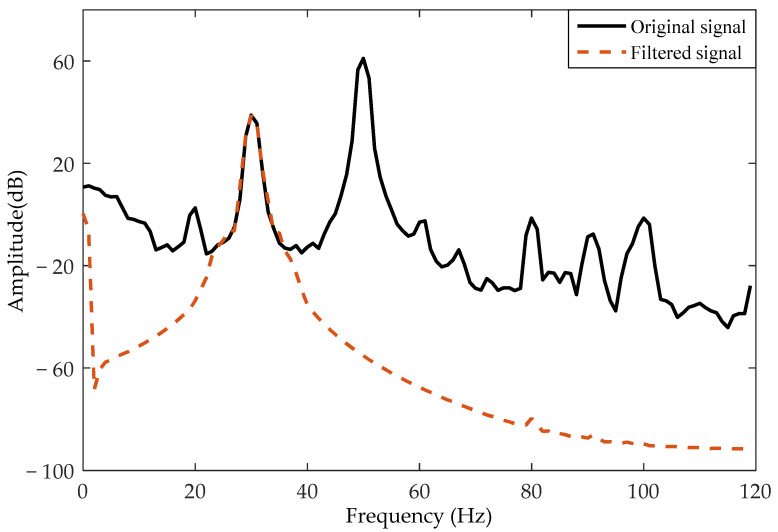
The spectrum of the output signal before and after filtering.

**Figure 14:**
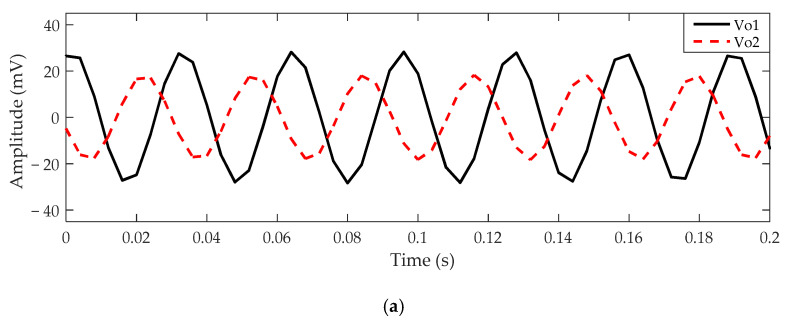

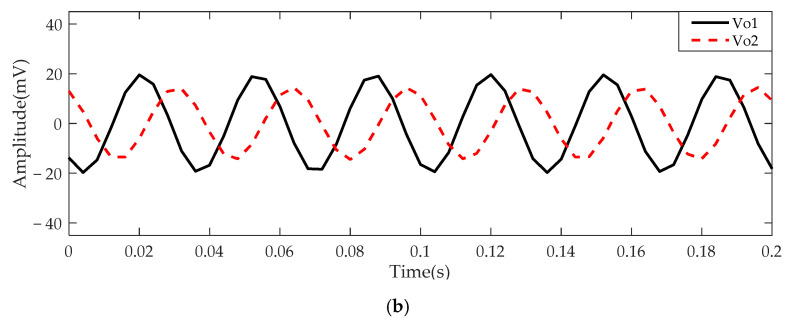
The output signals of *V*_o1_ and *V*_o2_: (**a**) obtained when the rotor rotates clockwise; (**b**) obtained when the rotor rotates anti-clockwise.

To remove the interferences from the rotation signal, the output signal for rotor rotation was processed with one band-pass filtering with center frequency 30 Hz and the quality factor 10. The spectrum curves before and after processing are shown in Figure 13. The processed output signals of the two electrodes for two rotation directions are shown in Figure 14. Through calculation by Equations (9)–(12), the phase difference between the two output signals was 125.9° for the clockwise direction and −108.2° for the counterclockwise direction. The amplitude difference between the two output signals might have been caused by the electrode position measurement error. In addition, compared with the simulated waveform, the amplitude of the experimental waveform was about one-fifth that of the simulated waveform. This might have been due to the charge amount carried by the rotor in the experiment being less than the simulation parameter. However, no matter whether the rotation was clockwise or anticlockwise, the phase relationship of the output signals of the two detection electrodes in the experiment was exactly the same as the simulation results. The phase relation deviation between the experimental results and the simulation result was about 10%, which might have been caused by actual measurement error.

**Figure 15:**
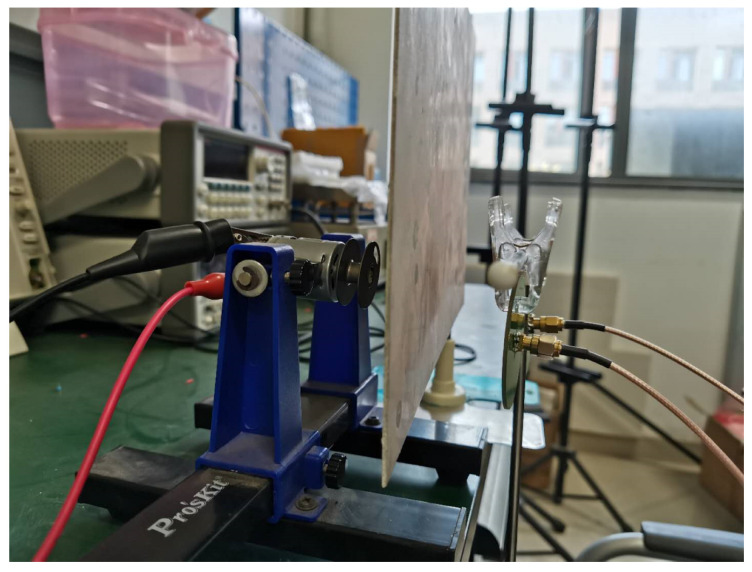
The experimental system with occlusion.

**Figure 16:**
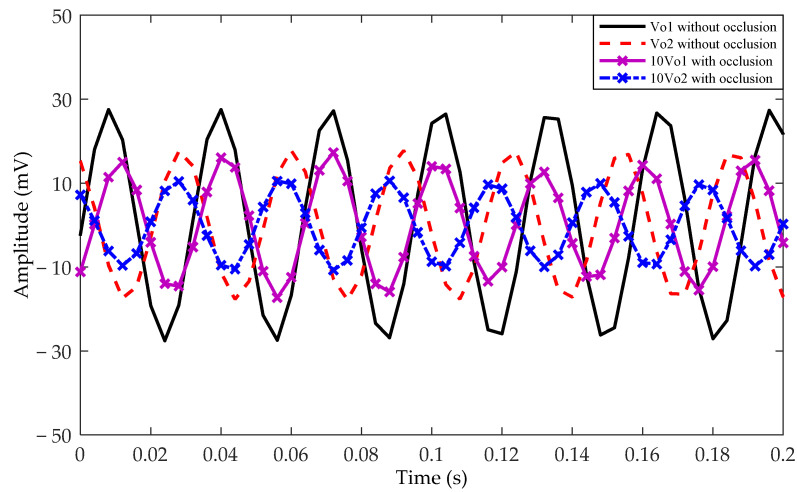
Comparison of the experimental waveforms with and without occlusion.

In order to show the ability of the proposed method for occlusion, a metal plate was placed between the electrodes and the rotor, as shown in Figure 15. The waveforms of electrodes for rotor rotation with occlusion are shown in Figure 16 by dotted line, where the amplitude of output signal with occlusion is enlarged by ten times. It can be seen that although the signal amplitude with occlusion is smaller to that without occlusion, the phase relationship between the two electrodes is unchanged regardless of whether there is occlusion or not. Therefore, the presence occlusion will not affect the recognition of the rotation direction.

### 3.3. Discussion

In the proposed method, the rotation direction can be identified by judging whether the phase difference is positive or negative. The theoretical phase difference between the two output signals mainly depends on the relative position of the two electrodes and the rotor. Generally, the greater the angle between the center of the rotor and the centers of the two electrodes, the larger the theoretical phase difference will be. In fact, the error tolerance of the calculation value of the phase difference is not the same for different theoretical phase difference. For example, when the theoretical phase difference of the system is 120°, the system can allow the calculation error to be less than 50%, which can be accurately judged as clockwise direction within the error range. However, when the theoretical phase difference of the system is 160°, the calculation error should be less than 12.5% to ensure correct identification of the rotor direction. The maximum calculation error of the proposed method is allowed to be 90° when the theoretical phase difference of the system is 90°. 

Table 1 shows the relative relationship between calculation error and sampling rate, number of sampling points, and rotor speed. Compared with the data from the first to the fourth groups, when the number of sampling points remains at 100, the calculation error gradually decreases from +50% to −2.6% as the sampling rate increases from 120 to 185 Hz. However, the data of groups 4–6 show that when the sampling rate is greater than 185 Hz, the error increases with the increase of the sampling rate. This is caused by the number of signal cycles used in the calculation of the correlation function not being enough, which is verified by the following data groups. Data from groups 6–8 show that when the sampling rate remains unchanged at 250 Hz, the calculation error decreases with the increase of sampling points. However, when the number of sampling points exceeds a certain value, the error will not decrease with the increase of the number of sampling points. Therefore, when setting system sampling rate and sampling points, it is necessary to take comprehensive consideration and select appropriate values. Small sampling rate or sampling points will increase the error, while large parameters settings will increase the complexity of the system and reduce the real-time performance.

In the case of the sampling rate at 250 Hz and the number of sampling points of 200, the results of data groups 7, 9, and 10 show that the calculation errors are not exactly the same for different theoretical phase differences, but the accuracy of direction recognition will not be affected. In the data groups 10–13, calculation errors were obtained for different rotor speeds. The results show that when the rotor speed exceeds 120 rotation per second, the system sampling rate of 250 Hz will be insufficient to ensure correct identification. 

Therefore, for the proposed rotor direction detection system based on charge induction, the appropriate electrode installation position can be selected first to ensure large calculation error tolerance by setting the theoretical phase difference around 90°. Then, according to the rotation speed of the measured rotor, the appropriate sampling rate and sampling points can be chosen to identify the rotor direction in real time. As for the statistical accuracy of the whole real-time system based on charge induction to identify rotor direction, we will conduct a large number of repetitive experiments in further research. 

## 4. Conclusions

In this paper, a novel method of rotor motion detection based on charge induction is proposed. Firstly, the charge detection model of rotor motion was illustrated, which showed that the detection signal *V_o_* is periodic due to the rotational motion of the rotor and the periodicity is equal to the time of one revolution of the rotor, although the signal amplitude decreases with the increase of distance between the electrode and the rotor. Secondly, the identification model of rotor direction was established with the phase difference between two detection signals obtained by two electrodes in front of the rotor, which was calculated using the correlation method. Finally, the detection circuit was designed, and the experiment was carried out. The experimental results show that the proposed method can effectively identify the noncontact rotor direction with and without occlusion. The accuracy of the method can be further improved by increasing the sampling rate and sampling points of the system. In summary, the proposed method is characterized by easy installation with only two electrodes in front of the rotor and can work well with occlusion as well as dust and electromagnetic interference. In the future, the accuracy of the rotor velocity of the proposed method will be studied, and the ability of antivibration from an industrial sewing machine will be investigated.

## Figures and Tables

**Table 1:** The relationship between error and sampling rate and sampling number.

Serial Number	Rotor Speed V(r/s)	Theoretical Phase Difference	Sampling Rate *f_s_* (Hz)	Number of Sampling Points *N*	Calculated Phase Difference	Error
1	30	60°	120	100	90°	+50%
2	30	60°	145	100	74.5°	+24.2%
3	30	60°	165	100	65.5	+9.2%
4	30	60°	185	100	58.4°	−2.6%
5	30	60°	200	100	54°	−10%
6	30	60°	250	100	43.2°	−28%
7	30	60°	250	200	57.6°	−4%
8	30	60°	250	300	57.6°	−4%
9	30	120°	250	200	129.6°	+8%
10	30	−160°	250	200	−172.8°	−8%
11	50	−160°	250	200	−144°	+10%
12	100	−160°	250	200	−144°	+10%
13	>120	−160°	250	200	Invalid	Invalid

## References

[1] Carvalho H., Rocha A., Monteiro J.L. An innovative device for bobbin thread consumption measurement on industrial lockstitch sewing machines. Proceedings of the 2004 IEEE International Conference on Industrial Technology. IEEE ICIT ’04..

[2] Liu C.H., Jywe W.Y., Tzeng S.C. (2004). Simple three-dimensional laser angle sensor for three-dimensional small-angle measurement. Appl. Opt..

[3] Liu X., Liu C., Pong P.W.T. (2018). Velocity Measurement Technique for Permanent Magnet Synchronous Motors Through External Stray Magnetic Field Sensing. IEEE Sens. J..

[4] Chen Q., Xue L., Rao H. (2017). Rotational speed measurement of ring spinning based on magnetic sensor. Meas. Sci. Technol..

[5] Todorovi A.S., Jevti M.D. (2010). Rotational Speed Measurement Using Induction Coil Sensor Inserted in the Magnetic Field of the Rotational Permanent Magnet. Facta Univ..

[6] Liu C., Xu Y., Liu J., Sun H., Kennel R. Rotational Speed Measurement Based on Avago ADNS-9800 Laser Mouse Sensor. Proceedings of the PCIM Europe 2016; International Exhibition and Conference for Power Electronics, Intelligent Motion, Renewable Energy and Energy Management.

[7] Didosyan Y.S., Hauser H., Wolfmayr H., Nicolics J., Fulmek P. (2003). Magneto-optical rotational speed sensor. Sens. Actuator A Phys..

[8] Qian J.Q., Cui Y., Xu P. (2008). The study for measuring rotor speed and direction with quadrant photoelectric detector. Measurement.

[9] Fabian M., Hind D.M., Gerada C., Sun T., Grattan K.T.V. (2018). Comprehensive Monitoring of Electrical Machine Parameters Using an Integrated Fiber Bragg Grating-Based Sensor System. J. Light. Technol..

[10] Iura H., Ide K., Hanamoto T., Chen Z. (2011). An Estimation Method of Rotational Direction and Speed for Free-Running AC Machines Without Speed and Voltage Sensor. IEEE Trans. Ind. Appl..

[11] Zhang X., Chen J., Wang Z., Zhan N., Wang R. (2012). Digital image correlation using ring template and quadrilateral element for large rotation measurement. Opt. Lasers Eng..

[12] Wang T., Yan Y., Wang L., Hu Y. (2018). Rotational Speed Measurement through Image Similarity Evaluation and Spectral Analysis. IEEE Access.

[13] Cheng P., Mustafa M.S.M., Oelmann B. (2012). Contactless Rotor RPM Measurement Using Laser Mouse Sensors. IEEE Instrum. Meas. Mag..

[14] Zhong J., Zhong S., Zhang Q., Peng Z. (2018). Measurement of instantaneous rotational speed using double-sine-varying-density fringe pattern. Mech. Syst. Signal Process..

[15] Zhong J., Zhong S., Zhang Q., Peng Z., Liu S., Yu Y. (2018). Vision-Based Measurement System for Instantaneous Rotational Speed Monitoring Using Linearly Varying-Density Fringe Pattern. IEEE Trans. Instrum. Meas..

[16] Guo J., Zhu C.a., Lu S., Zhang D., Zhang C. (2016). Vision-based measurement for rotational speed by improving Lucas–Kanade template tracking algorithm. Appl. Opt..

[17] Fan Y., Ji X. (2018). A Novel Rotation Speed Measurement Method Based on Surface Acoustic Wave. Acoust. Phys..

[18] Mata-Contreras J., Herrojo C., Martín F. (2018). Detecting the Rotation Direction in Contactless Angular Velocity Sensors Implemented With Rotors Loaded With Multiple Chains of Resonators. IEEE Sens. J..

[19] Wang L., Yan Y., Hu Y., Qian X. (2014). Rotational Speed Measurement through Electrostatic Sensing and Correlation Signal Processing. IEEE Trans. Instrum. Meas..

[20] Wang L., Yan Y., Hu Y., Qian X. (2015). Rotational Speed Measurement Using Single and Dual Electrostatic Sensors. IEEE Sens. J..

[21] Li L., Hu H., Qin Y., Tang K. (2019). Digital Approach to Rotational Speed Measurement Using an Electrostatic Sensor. Sensors.

[22] Wang S.F., Zhang L.M., Sun F., Dong Z.N., Yang X.W. (2019). A Recognition Method for Hand Motion Direction Based on Charge Induction. IEEE Sens. J..

[23] Wang G., Wang S.F., Zhang L.M., Sun F., Yang X.W. (2019). A New Light Control Method with Charge Induction of Moving Target. IEEE Sens. J..

[24] Trinks H., Haseborg J.L.T. (2007). Electric Field Detection and Ranging of Aircraft. IEEE Trans. Aerosp. Electron. Syst..

[25] Wu R., Tang Y., Li Z., Zhang L., Yan F. (2018). A novel high input impedance front-end for capacitive biopotential measurement. Med. Biol. Eng. Comput..

